# Non-classical monocytes in tissue injury and cancer

**DOI:** 10.18632/oncotarget.22584

**Published:** 2017-11-21

**Authors:** Ankit Bharat, Alexandra C. McQuattie-Pimentel, G.R. Scott Budinger

**Affiliations:** Ankit Bharat: Division of Thoracic Surgery, Department of Surgery and Division of Pulmonary & Critical Care Medicine, Department of Medicine, Northwestern University, Chicago, IL, USA

**Keywords:** non classical monocytes, tissue injury, cancer, neutrophils, disease pathogenesis

Monocytes can be subdivided into two ontologically related but functionally distinct subsets, namely classical (CM) and non-classical monocytes (NCM). While CM have been actively studied in inflammation and cancer for decades, only recently we started gaining insights regarding the role of NCM in disease pathogenesis. CM, after staying in the circulation for several days, can undergo apoptosis or differentiate into NCM. Steady state NCM “patrol” the vasculature via a mechanism that requires the fractalkine receptor CX3CR1, engulfing apoptotic endothelial cells and sensing danger signals coming from the tissue [1]. Our understanding of the functions of NCM was greatly enhanced by the recognition that the orphan nuclear receptor NR4A1 is necessary for the differentiation of CM into NCM [2]. Global *Nr4a1* knockout mice, which lack NCM, can, therefore, be used to determine whether they are causally linked to disease pathogenesis.

We recently used *Nr4a1*^-/-^ mice in a model of allogeneic lung transplantation to demonstrate a role for NCM in the recruitment of neutrophils. Specifically we found that NCM, anatomically located within the pulmonary vasculature, are retained in human and murine donor lungs at the time of transplantation [3, 4]. Transplantation of lungs from *Nr4a1*^-/-^ mice into wild-type recipients was associated with a marked reduction in neutrophil migration into the allograft following reperfusion, which was rescued by the adoptive transfer of wild-type NCM into *Nr4a1*^-/-^ donor lungs. Similarly, pharmacologically depleting NCM in the donor lungs prior to the transplantation with liposomal-clodronate reduced neutrophil trafficking. Using transcriptomic profiling and genetic murine knockouts, we showed that the NCM produced neutrophil chemoattractants including chemokine (C-X-C motif) ligand 2 (CXCL2) in a toll receptor dependent manner. Our findings bolster reports from other investigators demonstrating that depletion of NCM can ameliorate tissue injury in other models including arthritis, traumatic brain injury, and acute glomerulitis. A role for NCM has also been implicated in the pathogenesis of diseases such as neuromyelitis optica, primary biliary cirrhosis, atherosclerosis, vascular inflammation, coronary artery disease and cardiac failure. Contrastingly, reduced levels of NCM have been associated with increased susceptibility to Ebola virus.

There is also growing evidence implicating a role for NCM in cancer progression. Hanna et al. reported that NCM might prevent hematogenic spread of metastasis [5]. They found that NCM were concentrated in the vasculature near intravenously injected cancer cells and extravasated at tumor sites to engulf tumor material. Natural killer cell infiltration into lung metastatic lesions in models utilizing intravenous tumor cell injection or genetic induction of breast cancer was also impaired in *Nr4a1*^-/-^ mice, which resulted in increased disease burden. These phenotypes were improved in chimeric *Nr4a1*^-/-^ mice reconstituted with wild-type bone marrow. In contrast, in the setting of colorectal cancer, Jung et al. found that NCM promoted resistance to anti-VEGF therapy [6]. Targeted therapy against VEGF resulted in an upregulation of CX3CL1 in human colon cancer specimens and in murine models. Enhanced CX3CL1 expression promoted the recruitment of NCM to the vascular bed of the tumor, where they promoted recruitment of neutrophils. Both NCM and neutrophils inhibited T cell mediated anti-tumor immunity in part by secreting IL-10. Deleting NCM reduced neutrophil recruitment and IL-10 release in the tumor, resulting in improved anti-tumor immunity and efficacy of anti-VEGF therapy. Together these results suggest that NCM may play divergent roles in the different phases of tumor pathobiology and highlight the importance of understanding the mechanisms by which NCM contribute to anti-tumor immunity before they can be targeted for therapy. Such studies will likely be facilitated by the identification of an enhancer region in the NR4A1 promoter that is necessary for NCM differentiation but leaves NR4A1 expression and function in macrophages and other tissues intact. Thomas et al. recently used a mouse deficient in this enhancer region to confirm the importance of NCM in lung tumor metastases as described above [7].

In addition to their role in disease pathogenesis, several general biologic questions remain about the function of NCM. For example, while NCM do not express canonical markers of macrophages, their morphology, crawling behavior and function in efferocytosis have led some to suggest that they serve as endovascular macrophages. We now know that tissue resident macrophages in different organs are epigenetically programmed to differentiate from CM in response to cues provided by the local microenvironment [8]. Whether the distinct characteristic of the endothelium in different tissues imports tissue specific characteristics to NCM during homeostasis or injury is not known. Second, we know little about the fate of NCM recruited to sites of injury after activation. The precise mechanisms for the activation of NCM in response to injury are not known. We used unbiased profiling of the gene expression in NCM by RNA-Seq and found that several immune pathways, including sensing of double-stranded RNA were upregulated. Together with their dependence on MyD88/TRIF signaling, it may be possible that toll receptors, such as TLR3, may play a role in sensing of damage-associate molecular patterns by NCM. Better understanding of the functional biology of NCM and development of strategies to selectively deplete or augment their function might introduce strategies to manipulate them to ameliorate a variety of inflammatory diseases as well as cancer.
